# Electron paramagnetic resonance imaging for real-time monitoring of Li-ion batteries

**DOI:** 10.1038/ncomms7276

**Published:** 2015-02-09

**Authors:** M. Sathiya, J.-B. Leriche, E. Salager, D. Gourier, J.-M. Tarascon, H. Vezin

**Affiliations:** 1 Collège de France, 11 Rue, Place Marcelin Berthelot, 75231 Paris, France; 2 Sorbonne Universités, UPMC Univ Paris 06, 4 Place Jussieu, F-75005 Paris, France; 3 LRCS, CNRS UMR 7314, Université de Picardie Jules Verne, 80039 Amiens, France; 4 CNRS, CEMHTI (UPR3079), Université d’Orléans, 45071 Orleans, France; 5 Réseau sur le Stockage Electrochimique de l’Energie (RS2E), FR CNRS 3459, France; 6 PSL Research University Chimie Paristech, 11, Rue Pierre et Marie Curie, 75231 Paris, France; 7 University Lille Nord de France, CNRS, UMR 8516—LASIR, University Lille 1, F-59655 Villeneuve d’Ascq, France

## Abstract

Batteries for electrical storage are central to any future alternative energy paradigm. The ability to probe the redox mechanisms occurring at electrodes during their operation is essential to improve battery performances. Here we present the first report on Electron Paramagnetic Resonance *operando* spectroscopy and *in situ* imaging of a Li-ion battery using Li_2_Ru_0.75_Sn_0.25_O_3_, a high-capacity (>270 mAh g^−1^) Li-rich layered oxide, as positive electrode. By monitoring *operando* the electron paramagnetic resonance signals of Ru^5+^ and paramagnetic oxygen species, we unambiguously prove the formation of reversible (O_2_)^*n*−^ species that contribute to their high capacity. In addition, we visualize by imaging with micrometric resolution the plating/stripping of Li at the negative electrode and highlight the zones of nucleation and growth of Ru^5+^/oxygen species at the positive electrode. This efficient way to locate ‘electron’-related phenomena opens a new area in the field of battery characterization that should enable future breakthroughs in battery research.

## Abstract (web summary)

It is important as well as challenging to *in situ* probe redox mechanisms occurring at battery electrodes. Here, the authors develop an *in situ* electron paramagnetic resonance imaging technique and provide measurements on the nucleation growth of the anionic and cationic redox species at a battery electrode.

## Graphical abstract

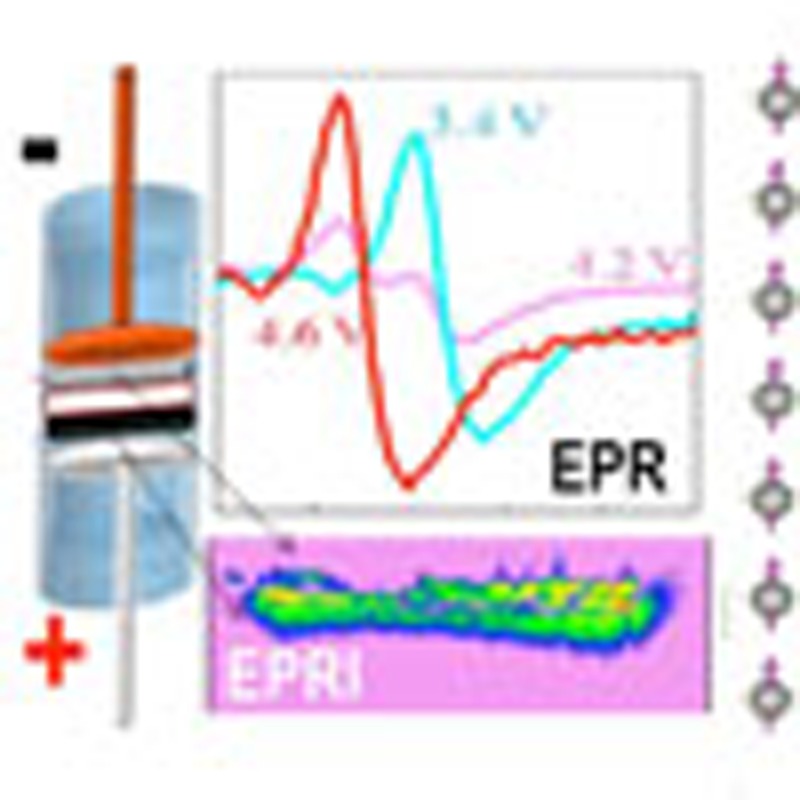

Rechargeable Li-ion batteries, because of their large energy density, have dominated the portable electronic market and are moving towards powering electric vehicles with other applications in load-leveling for large-scale storage of renewable energy^1^,^2^,^3^. For these applications to ever be commercially viable, batteries must continue to improve. Over the last two decades, the energy density of lithium batteries has nearly doubled. To continue the push forward in performance and energy density, the materials used in intercalation electrodes must be better understood, new concepts must be explored and the interface between the electrode and electrolyte must be optimized. For this to happen, basic structural/textural/physical information, which are central to the energy, power, safety and lifetime of battery systems, must be obtained and monitored as the battery system operates (*operando*).

Numerous *in situ* techniques have been developed over the years for the study of batteries as well as other material systems. The first *in situ* XRD technique to monitor electrochemical cycling was introduced by Chianelli *et al.*^4^ in 1978, to study Li_*x*_TiS_2_; it was then followed by a host of other techniques including Raman^5^, XANES^6^, NMR^7^, Mössbauer^8^, SEM^9^ and very recently *in situ* TEM microscopy^10^. The group of Clare Grey has shown that they can directly monitor the structural evolution of an insertion electrode or the growth of dendrites at the lithium electrode upon cycling^11^,^12^. Spectacular images of dendrites growing at the negative electrode–electrolyte interface upon cycling were observed very early by *in situ* SEM^9^ and the possible extension of this approach towards *in situ* TEM experiments has just been demonstrated in ref. 10 exploring the mechanistic of the Li uptake/removal in Si in an ionic liquid medium. To further accelerate the progress being made, we have reached a point where these analytical techniques must be pushed to their limits in terms of spatial, energy and time resolution with also a great demand for developing imaging techniques.

Classical positive electrodes for the Li-ion technology operate mainly via an insertion–deinsertion redox process involving cationic species. This is no longer the case with the development of Li-rich layered Li(Li_*x*_Ni_*y*_Co_*z*_Mn_*1−x−y−z*_)O_2_ phases, referred to as Li-rich NMC^13^,^14^,^15^, for which we previously demonstrated the redox activity occurring on the anionic network with the reversible formation of peroxo/superoxo-like groups (O^2−^→O_2_^*n*−^ where 3≥*n*≥1) to be responsible for their staggering capacities (280 mAh g^−1^). This was accomplished via a game-changing chemical approach (using Li_2_Ru_1−*y*_Sn_*y*_O_3_ as a model compound) coupled with X-ray photoemission spectroscopy (XPS) and electron paramagnetic resonance (EPR) measurements^16^,^17^. The ruthenate system was chosen because of its simple redox chemistry; Ru is the only redox-active cation, but preserves the structure and performance of the Li-rich NMC phases that contain three separate redox-active cations (Ni, Co and Mn). To date, EPR, which is a highly specific technique capable of detecting unpaired electrons or radicals^18^, has been very limitedly used within the battery community^19^,^20^,^21^,^22^,^23^,^24^,^25^. This study shows, however, that EPR is a powerful tool for characterizing the formation and disappearance of radical oxygen species during the cycling of a battery. EPR therefore offers unprecedented opportunities to study this new generation of high-capacity electrode materials and, to a certain extent, could enable electron-density visualization in a way analogous to the visualization of atoms with microscopy, provided that we can develop EPR imaging (EPRI).

EPRI is often used in the biological system for the detection of tumours by injecting spin labels^26^,^27^,^28^,^29^,^30^. However, EPRI is comparatively less commonly used than the widely popular magnetic resonance imaging (MRI) techniques because of the high specificity of EPRI towards unpaired electrons and technical challenges associated with the EPRI techniques. Extension of EPRI to Li-ion batteries brings new constraints: the electrochemical cell for the study should be made up of a material that is (i) compatible with all cell components (electrolytes, electrodes and so on), (ii) transparent to microwave radiation and (iii) inactive (silent) for EPR. In the present study, we develop a new electrochemical cell model that is compatible with all the aforementioned characteristics. The electrochemical cell (Fig. 1 inset) is designed on the basis of our cylindrical cell previously developed for *in situ operando* NMR^31^. Here we report the usage of the developed EPR cell for the *operando* EPR measurement of Li_2_Ru_0.75_Sn_0.25_O_3_ electrodes versus Li and its *in situ* imaging.

## Results

### *Operando* EPR experiments

**Figure 1:**
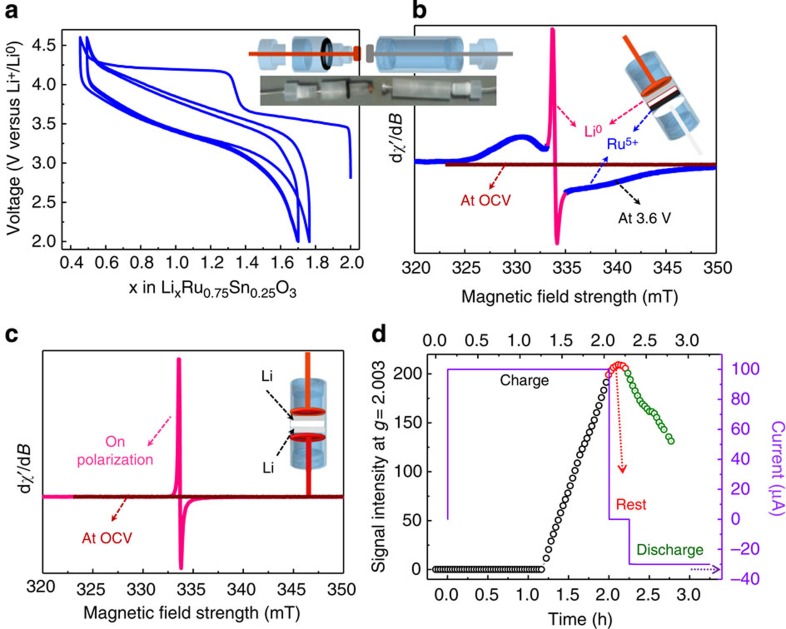
Cell design and electrochemical behaviour of the *in situ* EPR cell. (**a**) Electrochemical cycling performance of the cylindrical cell specially designed for *in situ* EPR measurements. Inset in **a**,**b** are the schematic of the cell configuration and the picture of the cell. The cell was assembled using Li_2_Ru_0.75_Sn_0.25_O_3_ as the positive electrode and Li metal foil as the negative electrode. (**b**) X-band EPR spectra of the Li_2_Ru_0.75_Sn_0.25_O_3_ versus Li half cell at OCV and after charging to 3.6 V. At OCV, all the components and cell parts are EPR-silent/inactive and there is no EPR signal. When the cell is charged to 3.6 V, a broad Ru^5+^ signal (blue part, *g*=2.0002) and a sharp Li metal signal (line marked in pink) was observed. (**c**) X-band EPR spectra of the Li versus Li symmetric cell. Bulk lithium did not show any EPR signal because of skin depth effect and a sharp signal because of deposited lithium particles is observed on polarization of the cell. (**d**) Change in intensity of Li metal signal on cycling the Li versus Li symmetric cell. The sharp EPR signal appears after polarizing the cell for 1 h by passing 100 μA. There is no change in signal intensity when the cell is kept at rest and small reduction in intensity is observed when the current is reversed.

Figure 1a shows the galvanostatic cycling profile of the Li_2_Ru_0.75_Sn_0.25_O_3_ electrode versus Li assembled in the specially designed EPR electrochemical cell. The cycling profile is analogous to the one previously obtained with these materials on either coin or Swagelok-type cells^17^. The electrochemical signature of the Li_2_Ru_0.75_Sn_0.25_O_3_ electrode versus Li presents two steps in the charging voltage curve followed by an S-shaped voltage variation during discharge. This EPR electrochemical cell was shown to sustain extended cycling, confirming its air tightness as well as the stability/compatibility of the Kel-F polymer towards electrolyte.

The *operando* EPR spectra collected at various steps in the electrochemical cycle are shown in Fig. 1b. Initially, the EPR spectrum is featureless, consistent with the fact that neither the active material Li_2_Ru_0.75_Sn_0.25_O_3_ (Ru^4+^ is EPR-silent) in the positive electrode nor the cell components contain unpaired electrons. However, the bulk Li foil at the negative electrode should exhibit a broad EPR signal because of unpaired electron spins at the top of the Li Fermi level. The featureless EPR spectrum/difficulty in observing the bulk Li EPR signal can be because of both the weak Pauli paramagnetism (low EPR intensity) and the skin depth effect, which limits the penetration of the microwave field to roughly a micrometre into the bulk (see Supplementary Note 1) and also because of strong dependence of the width of the metallic Li EPR signal with sample purity (the EPR signal width increases with increasing defects and impurity).

By charging the cell to 3.6 V we observed the appearance of a broad EPR signal centred at a value *g*=2.0002, smaller than the free electron spin value *g*_e_=2.0023. This signal is indicative of the presence of Ru^5+^, as unambiguously identified previously from the shape of the signals collected via *ex situ* measurements at both room and low temperatures (see Supplementary Note 2)^17^. It confirms the oxidation of EPR-silent Ru^4+^ (4d^4^) into EPR-active Ru^5+^ (4*d*^3^). During this charging step, we observed an additional very sharp and slightly distorted signal of 1.5 G line width, which is centred very close to the free electron ‘*g*’ value (*g*=2.0023). This sharp EPR signal is originating from the small metallic lithium aggregates that deposited on the negative lithium foil during the charging process; the signal intensity, line shape and line width are strongly dependent on the size and purity of the aggregates (See the description above for the featureless signal of bulk Li foil and Supplementary Note 1). For Li particles smaller than the skin depth (~1 μm), the EPR line was reported^32^,^33^ as symmetrical (*A*/*B*=1), with *A* and *B* being the amplitudes of the positive and negative parts of the signal, respectively. The fact that the EPR line is slightly distorted (*A*/*B*≈2.2) indicates that the size of Li particles (or the width of Li dendrites) is of the order of the skin depth or slightly larger.

We further tested this signal assignment by assembling a symmetric Li/electrolyte/Li electrochemical cell with metallic lithium as both positive and negative electrodes. No distinguishable Li EPR signal was detected for the as-made cell because of the reasons described above. Once we apply a continuous bias voltage (for example, *polarize the cell*), a sharp signal appears (Fig. 1c) with an intensity that continuously increases with increasing polarization time (Fig. 1d) and stabilizes as the bias voltage is turned off. Reversing the bias voltage triggers a decrease in the EPR signal, in contrast to the expectation of a constant signal. This difference most likely results from the time latency of removing Li agglomerates from the active EPR electrode and nucleating back on the opposite bulky electrode to form Li agglomerates with the critical dimension, for being EPR-detectable. Such a finding opens the possibility of using *in situ operando* EPR as a new and complementary tool to NMR for monitoring Li plating/stripping in real time, with the added benefit of seeing where dendrites nucleate with better resolution.

**Figure 2:**
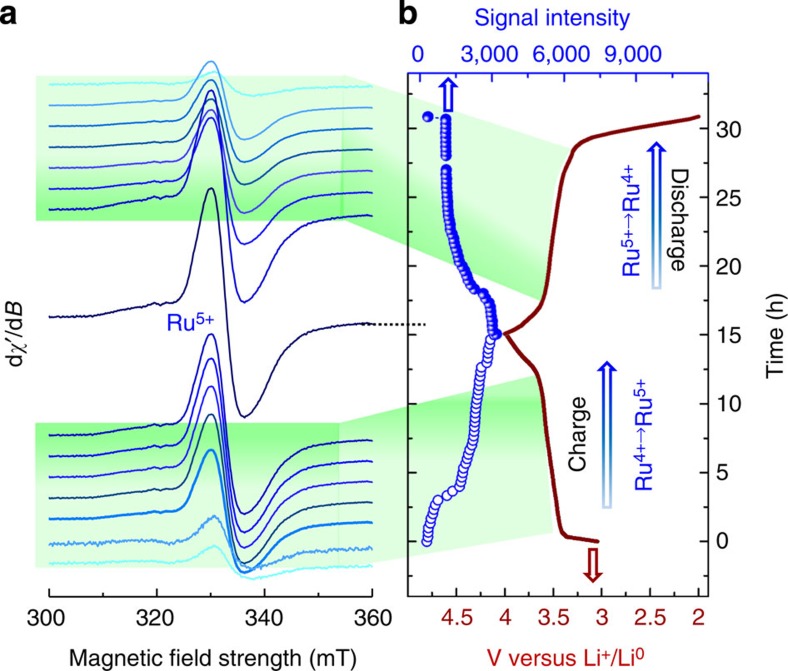
X-band EPR spectra of the Li_2_Ru_0.75_Sn_0.25_O_3_ versus Li half cell during cycling between 2 and 4 V. (**a**) X-band EPR spectra during charge from OCV to 4 V. The signal due to lithium metal particle is removed from the spectra for clarity reason. There is no EPR activity in the as assembled cell as well as after complete discharge (2 V) and is not shown in the Figure. (**b**) Corresponding electrochemical cycling of the cell. The intensity of the Ru^5+^ signal is followed at *g*=2.0002 and is shown by blue circles in **b**. The intensity of Ru^5+^ signal increases gradually on charge (blue open circles) and decreases on discharge (closed circles).

This *in situ* EPR technique is also very valuable to gain mechanistic insight of the Li-driven cationic–anionic redox processes occurring at the Li_2_Ru_0.75_Sn_0.25_O_3_ electrode by monitoring the evolution of the EPR signals *operando* as the Li/Li_2_Ru_0.75_Sn_0.25_O_3_ cell is continuously charged and discharged. The low-voltage region (2–4 V) was studied first. Figure 2 shows the collected EPR spectra, which for clarity are deconvoluted from the lithium metal signal so that the broad EPR signal centred at *g*=2.0002 because of Ru^5+^ ions can be nicely seen and analysed. We find (Fig. 2b) its intensity to continuously increase as the cell is charged to 4 V, and decrease to almost zero intensity when the cell is discharged back from 4 to 2 V. This implies a fully reversible cationic redox process over the first plateau as previously determined via combined *ex situ* EPR and XPS measurements^17^.

**Figure 3:**
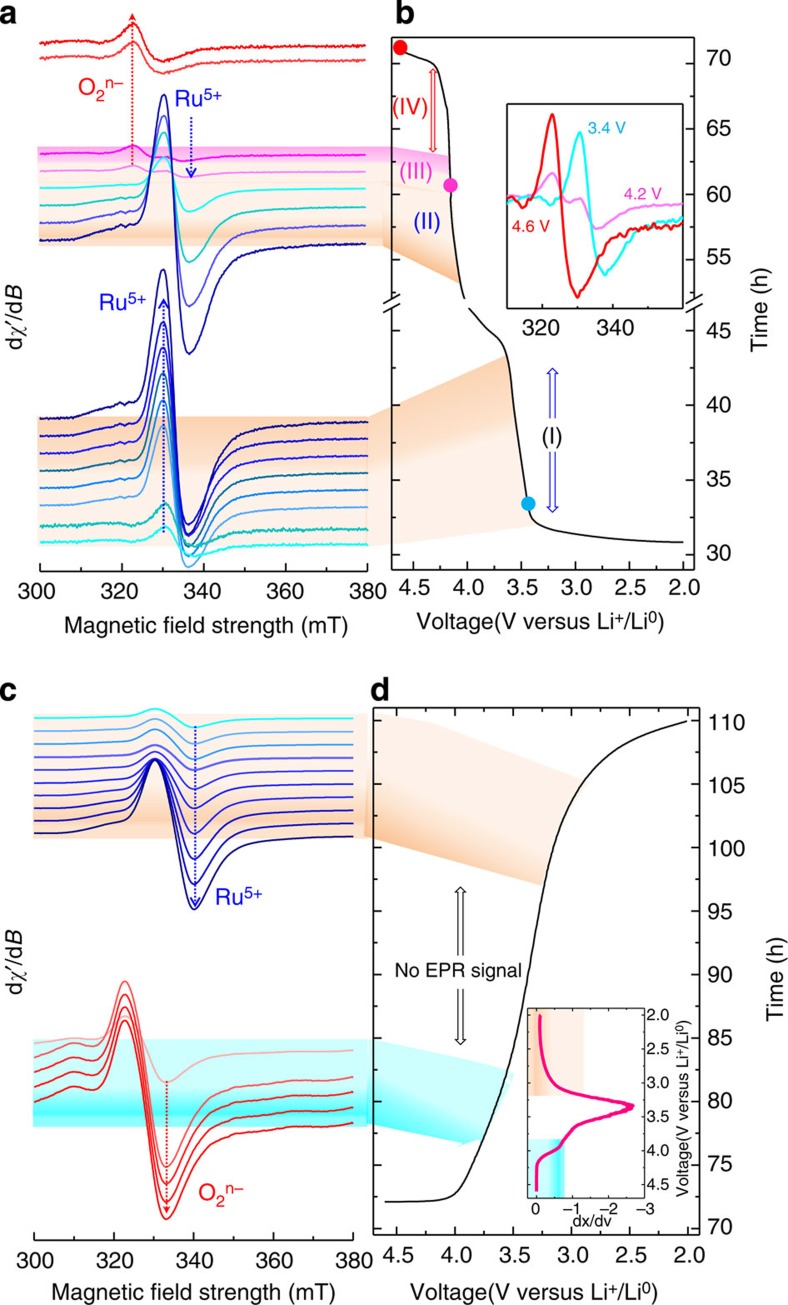
X-band EPR spectra of the Li_2_Ru_0.75_Sn_0.25_O_3_ versus Li half cell during cycling between 2 and 4.6 V. (**a**) X-band EPR spectra of Li_2_Ru_0.75_Sn_0.25_O_3_ versus Li half cell when the cell was charged from 2 to 4.6 V. The sharp EPR signal (*g*~2.003) due to lithium metal particle is removed from the spectra for clarity. There is no EPR activity at 2 V (not shown here) and the signal due to Ru^5+^ (*g*=2.0002) starts to appear on charge. The Ru^5+^ signal intensity increases continuously until the potential of the cell reaches 4 V (region I) and then decreased gradually (region II) on further charging. The signal due to Ru^5+^ and oxygen O_2_^*n*−^ (*g*=2.007) co-exist in Region III. Region IV shows the EPR signal associated only with oxygen O_2_^*n*−^ while no signal due to Ru^5+^ ion is observed. Corresponding cycling curve of the cell is shown in **b** and the inset shows the signal due to Ru^5+^ (3.4V), oxygen O_2_^*n*−^ (4.6V) and co-existence of both (4.2V) separately. (**c**) X-band EPR spectra of Li_2_Ru_0.75_Sn_0.25_O_3_ versus Li half cell during discharge from 4.6 to 2 V and corresponding cycling curve (**d**). Inset is the derivative plot of the cycling curve.

**Figure 4:**
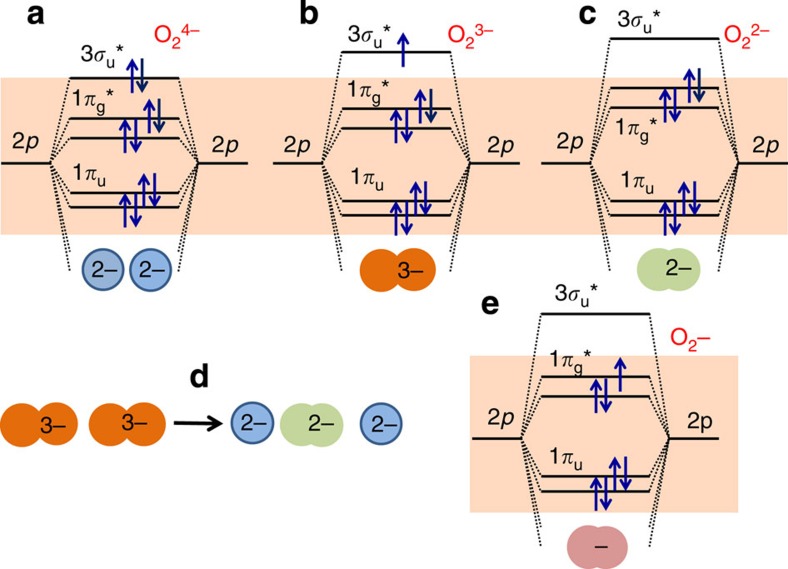
Schematic of the ‘O_2_’ molecular orbital diagram. The figure explains the nature of the oxygenated (O_2_)^*n*−^ species that we are discussing within the text and to highlight the fact that we are dealing with holes (or electrons) having antibonding characters. The orange area represents the oxygen-2*p* band. Note that (**a**) (O_2_)^4−^ molecular ion is not stable (number of bonding electrons=number of antibonding electrons), but it represents the corresponding diagram for a pair of neighbouring O^2−^ ions; (**b**) (O_2_)^3−^ and (**e**) (O_2_)^−^ possess single unpaired electrons hence their EPR activity, while (**c**) (O_2_)^2−^ is EPR inactive due to an even number of paired electrons. (**d**) Represents the condensation of two (O_2_)^3−^ (single hole traps) giving one (O_2_)^2−^ ion (two holes trapped at neighbouring oxygen sites).

To probe the overall redox processes in these high-capacity layered oxide electrodes, the electrochemical EPR Li/Li_2_Ru_0.75_Sn_0.25_O_3_ cell was cycled over the entire voltage range 2–4.6 V, over which cationic redox (Ru^4+^→Ru^5+^+e^−^) and anionic redox species (O^2−^→O_2_^*n*−^where 3≥*n*≥1) are expected. Consistent with previous *ex situ* experiments^17^, the intensity of the Ru^5+^ (*g*=2.0002) increases as the cell is charged to 4 V. On further charging beyond 4 V, there is a decrease in the Ru^5+^ signal and the appearance of a *new weak* signal (*g*=2.007) in the middle of the upper voltage plateau (see region III in Fig. 3a), which continuously grows before stabilizing as the cell reaches 4.6 V. This signal, whose line shape shows asymmetric broadening (inset Fig. 3b) and positive g-shift with respect to the free electron spin value (2.0023), was unambiguously assigned previously from *ex situ* room temperature and 4.2 K measurements to oxygenated species (for example, presence of holes in oxygen-2*p* orbitals)^17^,^34^. The formation of such paramagnetic species on charging was explained as the result of a reductive coupling mechanism triggered by the strong Ru(4*d*)–O(2*p*) orbital interaction. The anionic redox species that can enlist O_2_^3−^/O_2_^2−^/O_2_^−^ will be hereafter denoted as O_2_^*n*−^ where 3≥*n*≥1. Note that a pair of bulk O^2−^ ions, noted O_2_^4−^, has the electronic configuration π_g_*^4^σ*_u_^2^ (Fig. 4a) with complete filling of the antibonding orbitals. These filled antibonding *σ* orbitals correspond to the top of the oxygen-2*p* band. Consequently, the O^−^ ion formed upon oxidation is better described as an ion pair O_2_^3−^ of configuration (*π*_g_*^4^σ*_u_^1^), with a hole in the antibonding σ*_u_ orbital. The O–O bond length decreases and this has the effect to destabilize the antibonding σ*_u_ orbital and thereby stabilize the hole. Considering O_2_^*n*−^ species corresponding to the presence of holes in the antibonding orbital, it must be noticed that only O_2_^3−^ (*π*_g_*^4^σ*_u_^1^) and superoxo O_2_^−^ (*π*_g_*^3^σ*_u_^0^) species (Fig. 4b,e) possess an electronic spin *S*=1/2 and are thus EPR-active, while peroxo O_2_^2−^ (*π*_g_*^4^σ*_u_^0^; Fig. 4c) is EPR-silent (spin *S*=0). Thus, both O_2_^3−^ and O_2_^−^ are compatible with the EPR signal at *g*=2.007. The weakness of this signal can be explained by a partial condensation of two O_2_^3−^ pairs leading to EPR-silent O^2−^ and O_2_^2−^ species according to O_2_^3−^+O_2_^3−^→2O^2−^+O_2_^2−^ (Fig. 4d). Thus, the weak EPR line of oxygen species can be either because of the remaining O_2_^3−^ species that survive the condensation, or to superoxo O_2_^−^ species produced by oxidation of peroxo O_2_^2−^ species.

Turning to the discharge (Fig. 3c,d), we initially observe a decrease in the O_2_^*n*−^ signal to reach a voltage domain (3.5–3.2 V) over which the EPR spectra become featureless. Afterwards (<3.2 V), there is an abrupt resurgence of the Ru^5+^ signal whose intensity progressively decreases to nearly zero (Ru^5+^→Ru^4+^) as the cell voltage reaches 2 V. This further confirms an overall reversible process, although the reacting paths between the first charge and discharge are different. The EPR-silent voltage domain can be explained by the above model, enlisting the existence of several intermediate steps in the reduction of O_2_^*n*−^ species. Simply, during discharge there is at first the reduction in the EPR-active O_2_^3−^/O_2_^−^ species to give EPR-silent O_2_^2−^ and Ru^4+^ entities, which afterwards are replaced by active Ru^5+^ below ~3.3 V via an electron reorganization 2Ru^4+^+O_2_^2−^ →2Ru^5+^+2O^2−^. Then, Ru^5+^ is next reduced to Ru^4+^ consistent with the featureless EPR spectra collected at 2 V. Similar results were obtained when we duplicated the *in situ* operando EPR experiment (See Supplementary Fig. 1) proving the absolute reproducibility of the technique. These results further confirm the Li-driven reductive coupling mechanism that we proposed for these high-capacity electrode materials. Meanwhile, it is in very good agreement with the ^7^Li NMR spectrum of the fully charged electrode, observed previously to be very similar to the pristine Ru^4+^ material^31^, and it unambiguously explains why XPS spectra for the Ru-based high capacity electrodes constantly showed a downward rather than an upward shift of the Ru-3*d* core spectra as the samples were charged above 4 V.

*In situ operando* EPR, as described above, enables unprecedented insights into the fundamental understanding of complex redox mechanisms governing high-capacity electrodes. The chance of successfully turning such knowledge into design guidelines for practical electrodes will be enhanced by knowing where anionic redox species nucleate within the electrode; hence, the need to carry out spatially resolved measurements. This was the impetus to perform *in situ* EPRI of Li batteries, an approach that to the best of our knowledge had never previously been attempted.

### *In situ* EPRI

**Figure 5:**
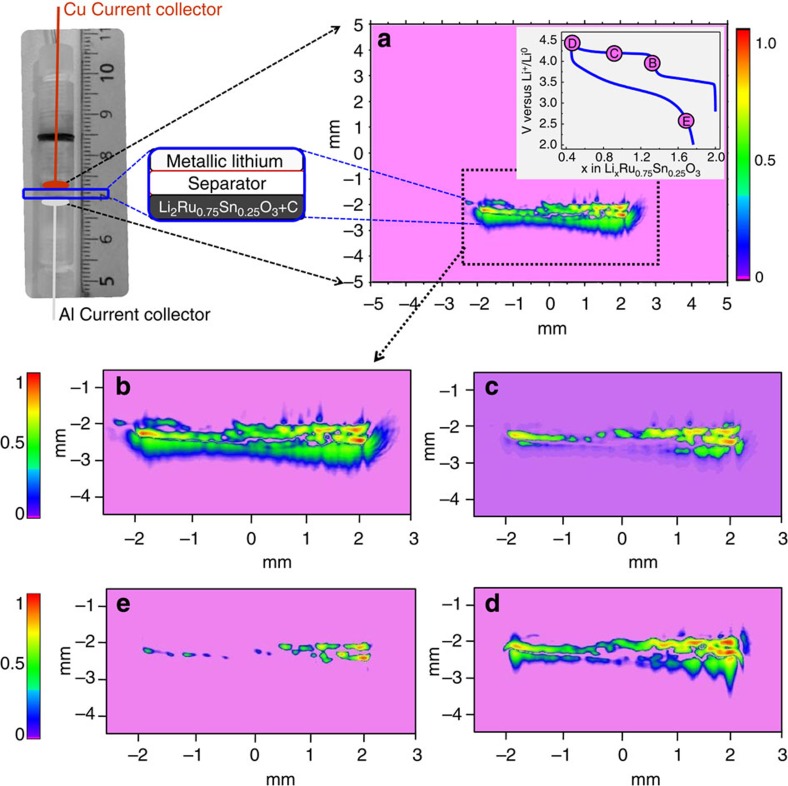
*In situ* EPR imaging. *In situ* EPR image of a Li/Li_2_Ru_0.75_Sn_0.25_O_3_ cell (**a**), which is cycled between 2 and 4.6 V with images taken at voltages defined by letters on the voltage-composition curve (inset A) by maintaining the cell on rest at these potentials for reasons indicated in the text. The first image as collected at 4 V is shown in **a** with a magnified amplitude in **b**. Images collected at 4.3, 4.6 and 2 V are shown in **c**–**e**, respectively. Colour bars have been placed on the left side as guide to the reader for visualizing the spin contribution with the extreme pink and red colours, representing the zone of no spin and high spin activity, respectively. The colour contrast changes of the electrodes through the various charging–discharging states are discussed on the text.

Next, we describe *in situ* EPR images obtained for Li/Li_2_Ru_0.75_Sn_0.25_O_3_ cells using ZY two-axis field gradients. Ideally, one would like to perform spectral/spatial *operando* imaging, that is, one spectrum recorded for each pixel of spatial distribution. Recording full spectral/spatial images with a spatial resolution of 20 μm per pixel is unfortunately not realistic in the present case, as it would take longer than 5 days to collect a single image. The complexity of EPRI depends further on both the number of signals and their respective sharpness. Luckily, the two different signals detected by CW-EPR for the Li_*x*_Ru_0.75_Sn_0.25_O_3_ samples have very different line widths of 1.5 G for the paramagnetic Li metal particles and between 40 and 50 G for the Ru^5+^ and the peroxo/superoxo species, respectively. This allows us to discriminate both contributions by encoding spatially the whole signal. Effectively, the gradients (175 G cm^−1^) used for encoding the two species will give a high resolution for the line with a width of 1.5 G (long transverse relaxation time), whereas for the broad line of 40–50 G (short transverse relaxation time) the resolution will be lower because of the smaller contribution of EPR-encoded signal. To extract both components, signal post-processing was applied in the deconvolution process. The high-resolution signal because of metallic Li was used as a probe to localize the battery. Moreover, for further simplification, EPRI was preferentially performed at voltage positions for which the positive electrode shows single Ru^5+^ or oxygen O_2_^*n*−^ signals. For better conveying the Li-driven changes in the electrode evolution, the collected EPRI images, summarized in Fig. 5, have been magnified to display a field of view of 5 mm, and show only ±2.5 mm from the centre of the resonator.

Figure 5a shows the EPR image of the cell collected at 4 V with the bottom and top parts due to the Li_2_Ru_0.75_Sn_0.25_O_3_ and Li metal electrode, respectively. The separator appears thinner than its real size on the acquired EPR image due to alignment issues during cell mounting as our current collectors connected to metal wires are not rigid enough. A magnified version of the EPR image (4 V) is displayed in Fig. 5b with on its left part a colour bar giving the normalized repartition of electronic spins with arbitrary red, green and blue colours indicating zones of high, medium and no spin concentrations, respectively. Both electrodes appear greenish at 4 V in agreement with the maximal amount of Ru^5+^ at the positive electrode and the already significant amount of Li deposits at the negative electrode. Nevertheless, the Li electrode shows additional red spots corresponding to Li aggregates of micrometric size (30 μm), indicating a non-homogeneous deposition of Li as commonly observed previously. This contrasts with the homogeneous colour of the positive electrode, which is indicative of a homogeneous distribution of the Ru^5+^ species. Charging the cell until 4.3 V (Fig. 5c) barely affects the spin concentration distribution at the negative electrode, but triggers a severe diminution of the spin contribution at the positive electrode. This is expected since, at that voltage, the Ru^5+^ disappears to give birth to the oxygen (O_2_)^*n*−^ signal (in accordance with Fig. 3a). When the cell is further charged to 4.6 V (Fig. 5d), there is no colour change at the negative electrode with the exception of more prominent reddish zones, indicative of an increase in metallic Li aggregate concentration, where dendrites may develop. The positive electrode, in contrast, becomes brighter because of the oxygen EPR signal, in accordance with our ‘Ru←O’ electron donation mechanism. Moreover, the distribution of oxygen O_2_^*n*−^ is heterogeneous at the micrometric level as indicated by different colour zones in contrast to the more homogenous distribution of the Ru^5+^ at 4 V. This information is of special interest as it outlines sluggish kinetics of the anionic redox process, a characteristic that will have to be overcome for practical applications. Finally, when the cell is discharged to 2 V (Fig. 5e), the acquired EPR image shows a residual signal of Li particles at the negative electrode. They are located in the high-Li concentration zones spotted on the 4- and 4.6-V images, which suggest that Li is not automatically stripped back from its high-concentration region on subsequent cycling as already documented^35^. There is no spin contribution at the positive electrode (pink colour) in agreement with the full reversibility of the Li insertion–deinsertion process into Li_2_Ru_0.75_Sn_0.25_O_3_.

For completeness, we extended this *in situ* EPRI to a symmetric Li/Li cell that we polarized as in Fig. 1c,d. We took images as a function of time and found evidence of nonuniform Li plating at the positively polarized electrode, but have so far not imaged dendrites, most likely because of limited resolution.

## Discussion

Today’s *in situ* EPRI is in its infancy and lacks high resolution. To increase EPR imaging resolution, two routes are being pursued: (1) drastic increase in the gradient strength up to 1 T cm^−1^; (2) use of an EPR microresonator with a higher B_1_ field to increase both sensitivity and resolution. While the first approach suffers from technical problems, the second one, as will be reported in a forthcoming paper, is feasible but requires the design of microbatteries for *operando* studies.

To conclude, we have reported that *operando* EPR is a powerful analytical tool for battery electrode characterization that provides unique information about redox processes in the anion network as well as the cation network. This information is vital for the optimization of next-generation Li-ion battery electrodes, as there is now an effective way to monitor superoxo/peroxo (O_2_^*n*−^) species that contribute to the high capacity of the Li-rich NMC electrodes. Moreover, it opens a multitude of exciting experiments to investigate the kinetics of the redox species as a function of current rates, potentials, resting times, electrolytes or temperatures, as well as studies of the complex and challenging issues surrounding SEI formation and parasitic reactions, all of which will be helpful in improving energy density, safety and lifetime of today’s batteries. This finding is timely regarding the number of technologies beyond Li ion that involve radical species such as Li–air, Li–S and Li–organic batteries with peroxides/superoxides, polysulfides or radical anions, respectively. Owing to its relative simplicity, we anticipate a rapid implementation of this technique to various battery technologies. Imaging is, by all means, the most attractive aspect of the EPR technique, although it is still restricted to micron resolution. We hope this work will accelerate the pace of EPR development within the battery community and attract worldwide experts to improve the spatial resolution of EPR imaging. Despite uncertainty about the size limits for these spatially resolved measurements, such local visualization could open a new area in the field of electrochemistry.

## Methods

### Synthesis of Li_2_Ru_0.75_Sn_0.25_O_3_

Stoichiometric amounts of RuO_2_ (Sigma-Aldrich, 99.9%) and SnO_2_ (Sigma-Aldrich, 98%) were homogenized with a 10% wt excess of Li_2_CO_3_ (Sigma-Aldrich, purity 99.0%) to compensate its volatilization at high temperature. The resultant mixture was heated at 800 °C for 24 h with intermediate grinding. Furnace heating and cooling rates were maintained at 2 °C min^−1^.

### Electrochemical insertion/extraction of Li^±^

*In situ* electrochemical EPR cells (described below) were assembled in an argon-filled glove box. Plastic Li_2_Ru_0.75_Sn_0.25_O_3_ electrodes were made according to the Bellcore Li-ion technology^36^, which consists of (i) preparing a slurry by mixing in acetone the active material, poly(vinylidene difluoride- hexafluoro propylene) (PVdF-HFP), C and Dibutyl phthalate (DBP) in a 56, 10, 10 and 24 weight % ratio, (ii) spreading the slurry on a coated Mylar foil, (iii) evaporating the acetone to obtain a self-handling film and (iv) removing the DBP by several washes (three times) in diethyl ether. Once oven-dried at 100 °C for 1 h the remaining plastic laminate is then punched in 3-mm-diameter disks weighting each nearly 4 mg. A Li metal disc is used as the negative electrode. Last, Whatman GF/D borosilicate glass fibre sheet of separator saturated with a LiPF_6_ solution (1 mol l^−1^) in a mixture of ethylene carbonate, propylene carbonate and dimethyl carbonate in 1:1:3 ratio by weight (LP100) was employed. Galvanostatic charge–discharge tests were conducted at 20 °C using a VSP system (Biologic S.A., Claix, France) operating in a galvanostatic mode. Unless otherwise specified, the cells were typically cycled between 4.6 and 2 V versus Li^+^/Li^0^ at 1 Li^+^ exchanged per Ru in 20 h (C/20).

### Electrochemical cell for operando EPR spectroscopy and *in situ* EPRI

The entire body of the cell was made up of a hydrophobic polymer Kel-F (polychlorotrifluoro ethane), which was previously checked for its electrolyte compatibility and EPR inactivity. For space constraints, determined by the slim design Low-Q TMHS resonator pertaining to the Bruker ELEXSYS E580 spectrometer, the outer and inner diameter of the cell was limited to 7 and 5 mm, respectively. Al and Cu disks welded to respective metal wires were used as the positive and negative current collectors. For Li-Li symmetric cell, Cu current collectors were used on both sides. Lastly, an EPR transparent home-made Teflon O-ring was placed between two parts to hermetically seal the cell (Fig. 1 (inset)).

### EPR spectra collection

EPR spectra were recorded at room temperature with a Bruker ELEXSYS E580 spectrometer, keeping the electrochemical cell under closed circuit during acquisition. Microwave power and modulation amplitude were, respectively, set to 5 mW and 5 G.

### EPR signal processing

Since the EPR signal during both charge and discharge is the overlap of two components, a broad one due either to the Ru^5+^ or oxo species and a narrow line due to metallic lithium, a deconvolution procedure was applied to extract the contribution of each component. Moreover, the narrow metallic lithium component was eliminated from the broad EPR components associated with Ru^5+^ and oxygen species by using a pseudomodulation algorithm at 20 G performed in origin 8.0 pro.

### EPR image collection

The signals were acquired with a field-of-view of 10 mm and gradient strength of 175 G cm^−1^. The electrochemical cell was kept under open circuit condition during image collection. The active part of the cell, nearly ~1 mm, was placed closest to the centre of the resonator to minimize the inhomogeneity of the B1 field of the resonator and to allow imaging of the whole electrochemical cell. The two-dimensional (2D) images were acquired with a size of 512 × 512 pixels resulting in a pixel size of 20 μm. Experiments were performed by continuously cycling the cells between 2 and 4.6 V at a C/20 rate with resting at various charge or discharge states to perform EPR imaging.

### EPR image processing

The image processing involves deconvoluting the whole signal acquired under a magnetic field gradient from the reference signal collected without gradient. To extract the narrow components from the broad line, we applied a pseudomodulation algorithm at 0.8 G combined with second derivative calculation. Both signals with and without gradient were computed and back-projected using Fourier Transform, giving the spatial distribution of metallic lithium. The same procedure was applied to obtain the broadline feature, with a 20-G pseudomodulation. Finally, both images were summed to obtain each component’s spatial distribution.

## Author contributions

E.S. conceived the first prototype of the electrochemical cell for NMR studies. J.-B.L., M.S. and J.-M.T. upgraded that prototype for the EPR experiments presented here. M.S. and J.-M.T. made the pristine materials and the electrochemical cycling of the samples. H.V. and M.S. carried out the *in situ* EPR experiments and H.V. analysed the data. All authors discussed the results. J.-M.T. and D.G. wrote the paper. All authors have given approval to the final version of the manuscript.

## Additional information

**How to cite this article:** Sathiya, M. *et al.* Electron paramagnetic resonance imaging for real-time monitoring of Li-ion batteries. *Nat. Commun.* 6:6276 doi: 10.1038/ncomms7276 (2015).

## Supplementary Material

Supplementary Information Supplementary Figure 1, Supplementary Notes 1-2 and Supplementary References
