# Supplementary material for: Electron paramagnetic resonance imaging for real-time monitoring of Li-ion batteries
Source: Nat Commun. 2015 Feb 9;6:6276. doi: 10.1038/ncomms7276 (PMC4347297; doi:10.1038/ncomms7276)
Supplement: Supplementary Information — Supplementary Figure 1, Supplementary Notes 1-2 and Supplementary References [file ncomms7276-s1.pdf]

Supplementary Information.

Supplementary Fig. 1: X-band EPR spectra of $Li_2Ru_{0.75}Sn_{0.25}O_3$ vs. Li half cells when the cell is charged from (a) Open circuit voltage (OCV) to 4 V, (b) 4 V to 4.6 V and (c) discharged to 2 V. The results obtained are in accordance with the previous *operando* EPR experiment described in the main text, hence proves the absolute reproducibility of the technique. There is no EPR activity in the as assembled cell. When the cell is charged, Ru^{5+} signal (g= 2.0002) started appearing and the intensity reaches a maximum when the cell voltage is raised to 4 V. On further charging the cell to 4.6 V, the signal due to oxygen species (O_2^{n-}) raise in intensity with the associated reduction in Ru^{5+} ion signal. On discharging the cell to 2 V, signal due to oxygen species appears till 4.3 V and there is no EPR activity in the cell after 4.3 V and the possible reasons are discussed in the main text.

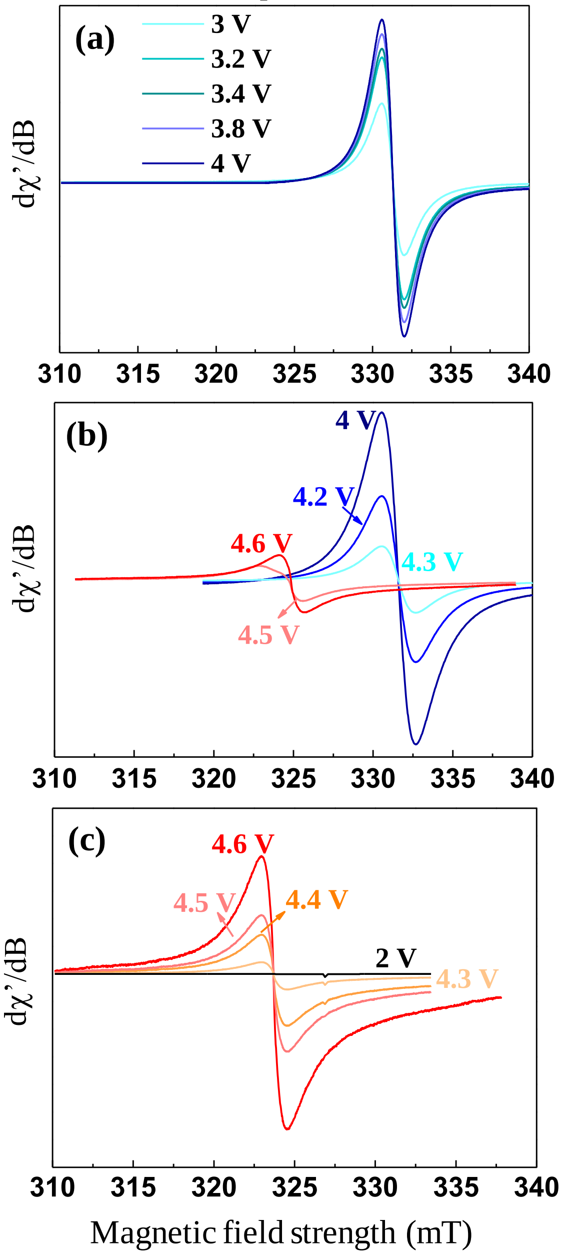

Supplementary note 1: The Li EPR signal

The appearance of the Li EPR signal from bulk metallic lithium is tricky and there are at least three possible reasons to account for such a difficulty. Firstly, the penetration depth of the microwave field in a metallic conductor is very small (on the order of a micrometer for Li) due to skin depth effect, so that a very small portion of the Li is probed. Secondly, it could be due to the fact that the Pauli paramagnetism of metal is much weaker than the Curie paramagnetism of isolated electron spins, by a factor of about $T/T_f \approx 5x10^{-3}$ for Li at room temperature, where $T_f = 5.5x10^{-4}$ K is the Fermi temperature of Lithium. Thirdly, we should bear in mind that the reported EPR line width ΔB of metallic lithium strongly depends on impurities and defects in the sample, with ΔB decreasing as defect and impurity content decreases. So the purity, disorder and morphology of the studied Li metal can influence its response in EPR.

When occasionally observed, the EPR signal for bulk Li is very distorted because of the skin depth effect with a distortion factor A/B=19, A and B being the amplitudes of the positive and negative parts of the signal, respectively.[1] For Li particles smaller than the skin depth (~1 μm), the EPR line is symmetrical (A/B=1). The fact that the EPR line is slightly distorted (A/B≈ 2.2) indicates that the size of Li particles (or the width of Li dendrites) is of the order of the skin depth or slightly larger. The line width ΔB=1.5 G is typical for “pure” metallic lithium.

Supplementary note 2: Assignment of the EPR line to Ru^{5+}

Needless to say that, the ‘g’ value is not sufficient to unambiguously assign the EPR signal to Ru^{5+}. We are relying here on our previous work which had consisted in collecting both room temperature and 4.2 K EPR spectra.[2] The 4 K spectra for the 4 V sample has shown clearly anisotropic signal with three components for the g factor (g_1=2.1, g_2=2.001 and g_3=1.90). However such signal is characteristic of a spin S=1/2 (d^1 configuration) instead of the spin S=3/2 expected for the d^3 configuration of Ru^{5+}. This is because the low symmetry around Ru splits the electronic ground state $^4A_2$ (t_{2g}^3) of Ru^{5+} into two levels characterized by spins components $m_s = \pm 1/2$ and $m_s = \pm 3/2$ (Kramers doublet). The strong spin-orbit coupling and the distorted environment of Ru gives an energy splitting (zero field splitting) between these two Kramers doublets that is larger than kT, so that only the lower doublet $m_s = \pm 1/2$ is populated. For this reason Ru^{5+} behaves as an effective S=1/2 system. This anisotropy of the g-matrix at low temperature vanishes at room temperature, giving a broad single line at an averaged g-value $g = (g_1 + g_2 + g_3)/3 = 2.0003$ *(See figure below issued from our Nature Materials paper)*. This motional averaging is likely due to the delocalization of the electrons in the Ru-4*d* band. So from such reasons we could unambiguously infer the signal observed at 4 V as due to Ru^{5+}.

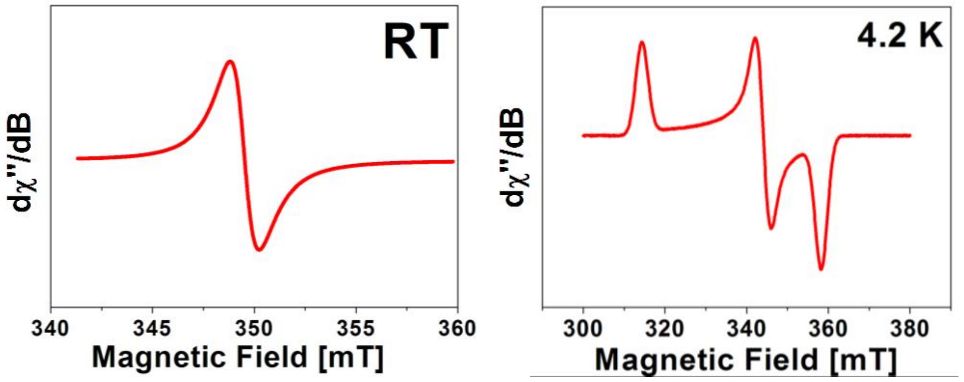

Supplementary references:

1. Feher, G. & Kip, A. F. Electron Spin Resonance Absorption in Metals. I. Experimental. *Phys. Rev.* **98**, 337-348 (1955).

2. Sathiya, M., *et al.* Reversible anionic redox chemistry in high-capacity layered-oxide electrodes. *Nat. Mater.* **12**, 827-835 (2013).
